# Global gene expression profiling of JMJD6- and JMJD4-depleted mouse NIH3T3 fibroblasts

**DOI:** 10.1038/sdata.2016.22

**Published:** 2016-04-12

**Authors:** Yu-Jie Hu, Anthony N. Imbalzano

**Affiliations:** 1 Department of Cell and Developmental Biology, University of Massachusetts Medical School, Worcester, Massachusetts 01655, USA

**Keywords:** Histone post-translational modifications, Microarray analysis, RNAi, Cell growth

## Abstract

Emerging evidence suggests Jumonji domain-containing proteins are epigenetic regulators in diverse biological processes including cellular differentiation and proliferation. RNA interference-based analyses combined with gene expression profiling can effectively characterize the cellular functions of these enzymes. We found that the depletion of Jumonji domain-containing protein 6 (JMJD6) and its paralog protein Jumonji domain-containing protein 4 (JMJD4) individually by small hairpin RNAs (shRNAs) slowed cell proliferation of mouse NIH3T3 fibroblasts. We subsequently performed gene expression profiling on both JMJD6- and JMJD4-depleted mouse NIH3T3 fibroblasts using the Affymetrix GeneChip Mouse Exon 1.0 ST Array. Here we report the gene profiling datasets along with the experimental procedures. The information can be used to further investigate how JMJD6 and JMJD4 affect gene expression and cellular physiology.

## Background & Summary

Jumonji domain-containing proteins are iron- and 2-oxoglutarate-dependent oxygenases that act on diverse substrates including proteins, nucleic acids and small molecules^1,2^. These enzymes either hydroxylate or demethylate their substrates in an oxygen-dependent manner. Many JmjC domain proteins play a key role in the epigenetic regulation of mammalian development and of diseases such as cancer^2,3^.

Phylogenetic analysis classifies the Jumonji domain containing proteins into several subgroups^3^. One subgroup comprises the asparaginyl hydroxylase FIH^4,5^, the ribosomal hydroxylases MINA53 and NO66 (ref. 6) , the lysyl hydroxylase JMJD4 (ref. 7), the lysine demethylase/hydroxylase JMJD5 (refs 8,^9^) , the lysyl hydroxylase/arginine demethylase JMJD6 (refs 10,^11^) , the tRNA hydroxylase TYW5 (ref. 12), and the enzymatically uncharacterized proteins JMJD8 and HSPBAP1. Although these enzymes share the conserved JmjC domain with the histone lysine demethylases, they generally lack chromatin-binding domain, indicating potential functions other than modifying histones.

Among the members of this functionally diverse subgroup, JMJD6 was recently characterized as a crucial regulator for gene expression at the level of histone modification^10,13^, transcriptional elongation^14^ and RNA splicing^11,15,16^. *Jmjd6*-deficient mice show various developmental defects and perinatal lethality^17,18^. Moreover, JMJD6 is required for angiogenesis^15^, adipocyte differentiation^19^ and T cell proliferation^20^. Elevation of JMJD6 expression has been observed in breast^21,22^, lung^23^, colon^24^, and oral cancer^25^. Depletion of JMJD6 by RNA interference reduced the proliferation of human cancer cell lines^21,24^. Therefore, JMJD6 is essential for cellular proliferation and differentiation. In contrast, the paralog protein JMJD4 that shares 34% sequence identity with JMJD6 was shown to be involved in translational termination by hydroxylating the translational termination factor eRF1 (ref. 7). Other biological functions of JMJD4 have not been demonstrated.

We conducted a loss-of-function study using an RNA interference approach to reduce the levels of endogenous JMJD6 and JMJD4 in proliferating mouse NIH3T3 fibroblasts. We found that depletion of either JMJD6 or JMJD4 alone significantly reduced cell proliferation. In order to determine whether and how these JmjdC domain proteins affect gene expression on a global scale, we performed gene expression profiling on the JMJD6 and JMJD4 knockdown NIH3T3 cells using the Affymetrix Exon Arrays. In this report, we provide a detailed description of the gene expression profiling datasets.

## Methods

**Figure 1:**
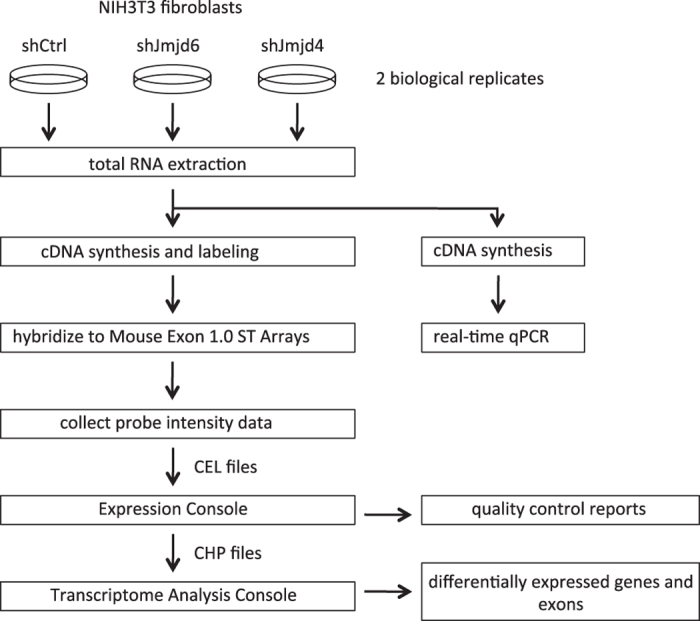
The scheme of the experimental design.

The scheme of the experimental procedures is presented in Fig. 1.

### Cell culture

The mouse NIH3T3 fibroblast cell line was maintained in DMEM high glucose medium (Invitrogen) containing 10% calf serum (Sigma) and 100 U ml^−1^ penicillin/streptomycin (Invitrogen). Human 293T cells were maintained in DMEM high glucose medium containing 10% fetal calf serum (Sigma) and 100 U ml^−1^ penicillin/streptomycin (Invitrogen). To evaluate cell proliferation, 1×10^5^ cells were seeded in 6-well plates, and the number of viable cells was counted every other day with a hemocytometer.

### Plasmid construction and virus transduction

The preparation of small hairpin RNA (shRNA) lentiviral constructs was performed as previously described (Campeau, 2009). The shJMJD6 target sequence is 5′-GCTGACACCCAGAGAACAA-3′. The shJMJD4 target sequence is GAAGAATTCTGGAGAGCAT. The control shRNA sequence is TGGCGGCGAGTGAAGTACGTGATAA. The shRNA lentiviral constructs were co-transfected with pLP1, pLP2 and pVSVG packaging vectors into 293T cells with Lipofectamine 2000 reagent (Invitrogen). The viral supernatant was harvested after 48 h incubation and filtered through a 0.45 μm syringe filter (Millipore). To infect NIH3T3 cells, one million cells were incubated with 2 ml of the filtered viral supernatant supplemented with 4 μg ml^−1^ of polybrene (Sigma) for 48 h. The infected cells were subsequently selected in media containing 2.5 μg ml^−1^ puromycin (Invitrogen) for 7 days.

### RNA isolation and real-time qPCR analysis

One million cells were plated overnight in 10 cm culture dishes. The cells were washed with 5 ml cold PBS and then lysed directly in the culture dish by adding 1 ml of TRIzol reagent (Invitrogen). Total RNA was isolated from TRIzol lysates using RNeasy Mini Kit (Qiagen) according to the manufacturer’s instructions. An on-column DNAse digestion step is included in this protocol. cDNA was prepared from 1 μg of total RNA by Superscript III reverse transcriptase kit (Invitrogen). Quantitative PCR was performed on a StepOne Plus real-time PCR machine with Fast SYBR Green Master mix (Applied Biosystems). The relative gene expression levels were calculated as 2^ (Ct_*Eef1a1*_−Ct_*gene*_) and were normalized to the scrambled shRNA control as indicated. The primers are listed below:

*Eef1a1* (forward): 5′-AGCTTCTCTGACTACCCTCCACTT-3′

*Eef1a1* (reverse): 5′-GACCGTTCTTCCACCACTGATT-3′

*Jmjd6* (forward): 5′-GTTCCAGCTCGTCAGACTCG-3′

*Jmjd6* (reverse): 5′-TGCCCCTAAGACATGACCAC-3′

*Jmjd4* (forward): 5′-TCCTGCTGGAATGTCGCACCTGT-3′

*Jmjd4* (reverse): 5′-ACCCCAAATAGGGACCGGAGGC-3′

### Microarray analysis

The RNA samples were processed at the Genomics Core Facility of UMass Medical School. RNA quantity and quality were assessed using an Agilent Bioanalyzer 2100. cDNA was synthesized from 500 ng RNA using the Ambion WT kit (Life Technologies) and the single stranded cDNA was prepared using the Affymetrix GeneChip WT Terminal Labeling Kit (Affymetrix) according to the manufacturer’s instructions. The labeled mix was hybridized to GeneChip Mouse Exon 1.0 ST Array (Affymetrix) in the Gene Chip Hybridization Oven 640 overnight. Probe intensities were measured using the Affymetrix GeneChip Scanner 3000 7G. The probe cell intensity data (CEL) from GeneChip Mouse Exon 1.0 ST Arrays was analyzed in the Affymetrix Expression Console software to generate CHP files using the Robust Multichip Analysis (RMA)-sketch algorithm workflow. The transcript structure confidence levels for both gene and exon level analyses were set as Core, which limits analysis to exon-level probe sets that map to BLAT alignments of mRNA with annotated full-length coding sequence (CDS) regions. Differentially expressed genes and exons were identified by Transcriptome Analysis Console (TAC) 3.0 software (Affymetrix).

## Data Records

Gene expression profiling on RNA samples collected from the control shRNA-treated (shCtrl_R1 and shCtrl_R2), JMJD6 knockdown (shJMJD6_R1 and shJMJD6_R2), JMJD4 knockdown (shJMJD4_R1 and shJMJD4_R2) cells was performed using GeneChip Mouse Exon 1.0 ST Arrays. Two biological replicates were performed. Both gene level and exon level expression were analyzed. All samples and datasets are described in Table 1. The primary data are available at the NCBI Gene Expression Omnibus (GEO) under the accession numbers(Data Citation 1).

## Technical Validation

### Confirmation of the shRNA-mediated knockdown

**Figure 2:**
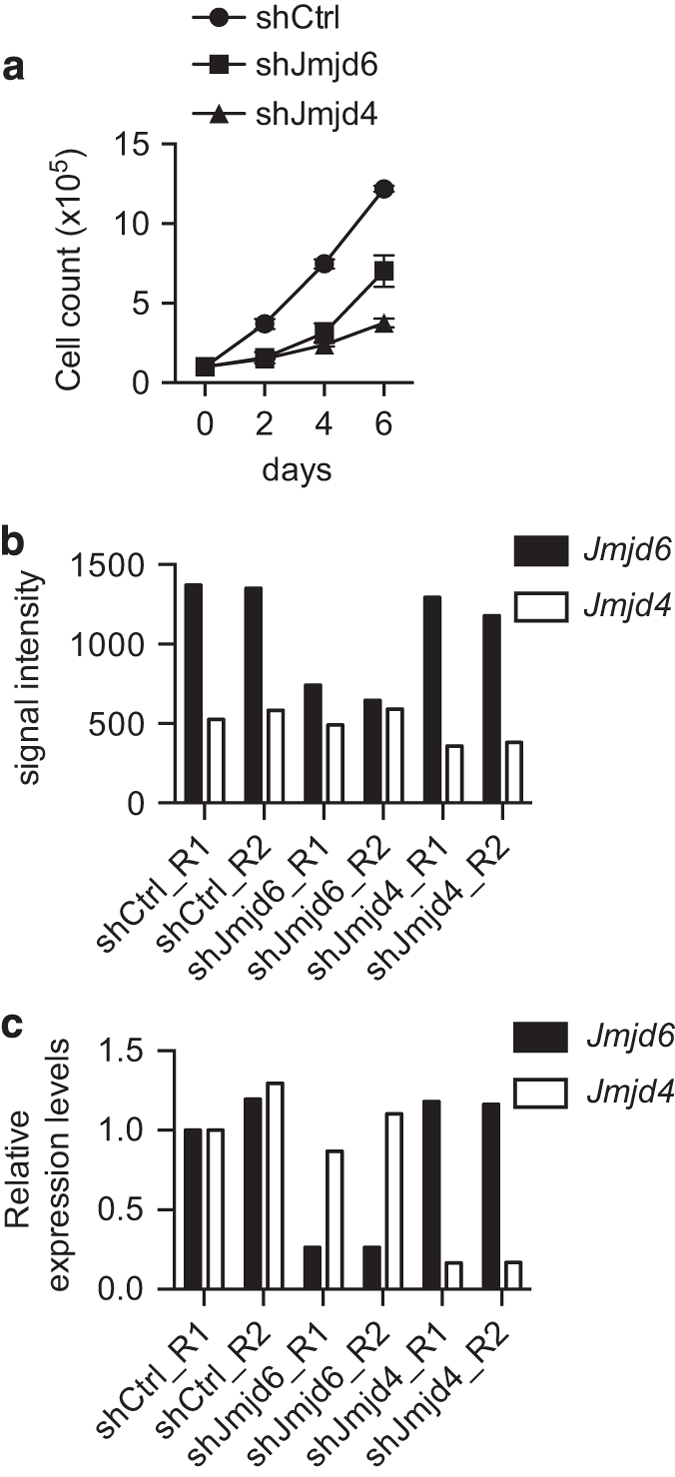
The effects of shRNA-mediated knockdown of JMJD6 or JMJD4 on mouse NIH3T3 fibroblast proliferation. (**a**) The cell proliferation curve of the control shRNA-treated (shCtrl), JMJD6 knockdown (shJMJD6), and JMJD4 knockdown (shJMJD4) mouse NIH3T3 fibroblasts. (**b**) The signal intensity of expression from the *Jmjd6* and *Jmdj4* genes from each individual array. (**c**) The relative expression levels from the endogenous *Jmjd6* and *Jmdj4* genes in each sample were determined by real-time qPCR analysis. The value of the shCtrl_R1 sample was set as 1.

The shRNA-mediated knockdown reduced cell proliferation is shown in Fig. 2a. The signal intensity values of expression from the *Jmjd6* and *Jmjd4* genes in the microarray datasets are shown in Fig. 2b. The expression levels from the endogenous *Jmjd6* and *Jmjd4* genes were confirmed by real-time qPCR and are shown in Fig. 2c.

### Quality control of microarray data

**Figure 3:**
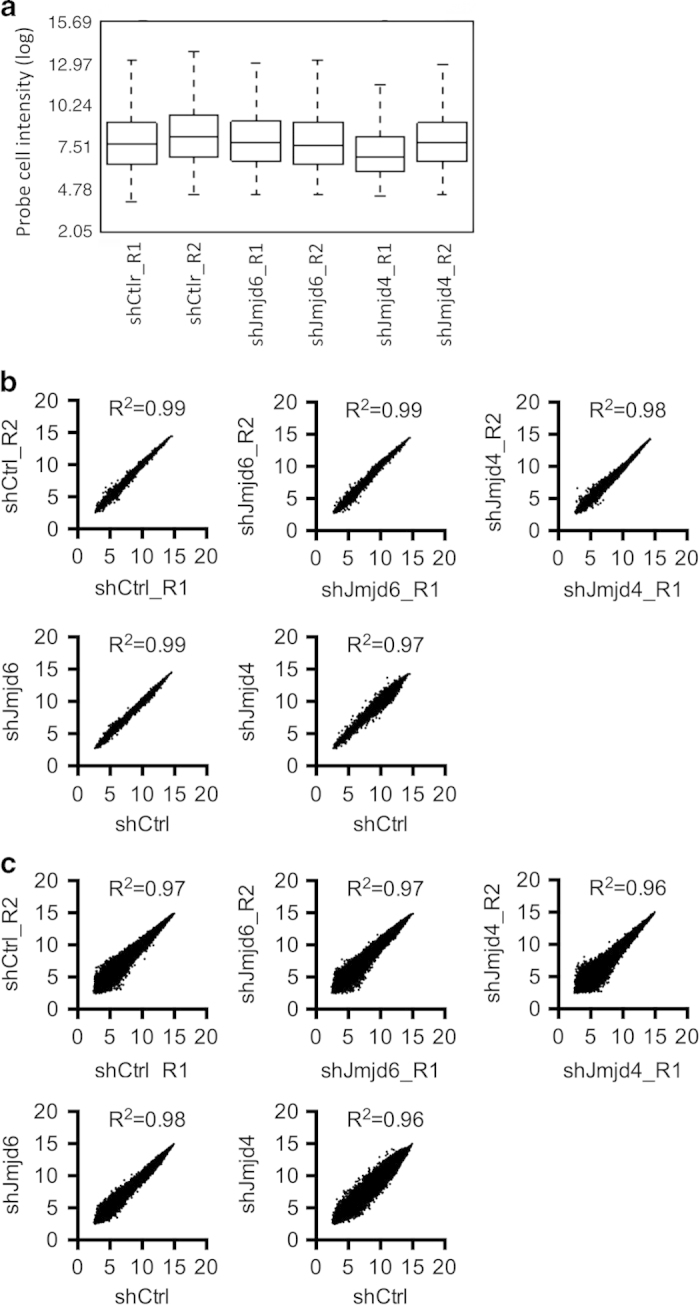
Quality assessment of the microarray datasets. (**a**) The box plot of feature probe cell intensity values from each individual array. (**b**) The scatter plots of signal intensity from each sample pair at the level of gene expression. (**c**) The scatter plots of signal intensity from each sample pair at the level of exon expression. R^2^: the squared Pearson correlation coefficient.

The probe cell intensity values in each individual array were generated from the Affymetrix Expression Console software and are presented in the box plot (Fig. 3a). The data indicate that the probe cell intensities from the individual arrays were similar. The reproducibility of the microarray results is shown by the correlation analyses on the biological replicates. The correlation between the samples at the gene level (Fig. 3b) and at the exon level (Fig. 3c) are presented in the scatter plots with the squared Pearson correlation coefficient (R^2^). All pairs of biological replicates have very high correlation (R^2^≥0.98 for gene level analysis; R^2^≥0.96 for exon level analysis). Knockdown of JMJD4 has more profound impact on gene expression than knockdown of JMJD6 (R^2^=0.97 versus R^2^=0.99 for gene level analysis; R^2^=0.96 versus R^2^=0.98 for exon level analysis).

## Usage Notes

The full quality control report can be accessed using the Affymetrix Expression Console software. The differentially expressed genes and exons can be identified using the Affymetrix Transcriptome Analysis Console software.

## Additional Information

**How to cite this article:** Hu, Y.-J. & Imbalzano, A. N. Global gene expression profiling of JMJD6- and JMJD4-depleted mouse NIH3T3 fibroblasts. *Sci. Data* 3:160022 doi: 10.1038/sdata.2016.22 (2016).

## Supplementary Material



## Figures and Tables

**Table 1:** Sample and Dataset Descriptions.

**Sample name**	**Cell line**	**Description**	**Dataset**
shCtrl_R1	NIH3T3	Control shRNA-treated, biological replicate 1, gene level analysis	GSM2037236
shCtrl_R1	NIH3T3	Control shRNA-treated, biological replicate 1, exon level analysis	GSM2037237
shCtrl_R2	NIH3T3	Control shRNA-treated, biological replicate 2, gene level analysis	GSM2037238
shCtrl_R2	NIH3T3	Control shRNA-treated, biological replicate 2, exon level analysis	GSM2037239
shJMJD6_R1	NIH3T3	JMJD6 shRNA-treated, biological replicate 1, gene level analysis	GSM2037240
shJMJD6_R1	NIH3T3	JMJD6 shRNA-treated, biological replicate 1, exon level analysis	GSM2037241
shJMJD6_R2	NIH3T3	JMJD6 shRNA-treated, biological replicate 2, gene level analysis	GSM2037242
shJMJD6_R2	NIH3T3	JMJD6 shRNA-treated, biological replicate 2, exon level analysis	GSM2037243
shJMJD4_R1	NIH3T3	JMJD4 shRNA-treated, biological replicate 1, gene level analysis	GSM2037244
shJMJD4_R1	NIH3T3	JMJD4 shRNA-treated, biological replicate 1, exon level analysis	GSM2037245
shJMJD4_R2	NIH3T3	JMJD4 shRNA-treated, biological replicate 2, gene level analysis	GSM2037246
shJMJD4_R2	NIH3T3	JMJD4 shRNA-treated, biological replicate 2, exon level analysis	GSM2037247
